# Supplementary material for: Ultrasonic Deep Brain Neuromodulation in Acute Disorders of Consciousness: A Proof-of-Concept
Source: Brain Sci. 2022 Mar 23;12(4):428. doi: 10.3390/brainsci12040428 (PMC9032970; doi:10.3390/brainsci12040428)
Supplement: Supplementary file 1 [file brainsci-12-00428-s001.zip › brainsci-1601136-supplementary.pdf]

#	CRSR_AF	CRSR_VF	CRSR_MF	CRSR_OF	CRSR_C	CRSR_Ar	CRSR_Tot
**1**	3	5	4	1	0	2	15
	2	4	4	1	1	2	14
	**LIFUP_1**						
	2	4	4	1	0	2	13
	3	5	5	2	1	1	17
**2**	0	0	3	1	0	1	5
	0	0	3	1	0	1	5
	**LIFUP_1**						
	0	0	3	1	0	1	5
	0	0	3	1	0	1	5
**3**	3	3	3	1	0	1	11
	3	3	1	1	0	1	9
	3	3	5	1	0	2	14
	**LIFUP_1**						
	3	2	5	1	0	1	12
	3	2	5	1	0	1	12
	3	4	5	1	0	2	15
	2	2	5	1	0	2	12
	**LIFUP_2**						
	2	4	5	1	0	2	14
	2	4	5	1	0	2	14
	4	5	6	3	2	3	23
**4**	0	0	1	0	0	0	1
	0	0	1	0	0	0	1
	**LIFUP_1**						
	0	0	2	1	0	0	3
	0	0	0	0	0	0	0
	0	0	0	0	0	0	0
	**LIFUP_1**						
	2	0	2	1	0	1	6
	2	0	2	1	0	1	6
	2	0	2	1	0	0	5
**5**	1	0	1	1	0	1	4
	1	0	2	1	0	0	4
	**LIFUP_1**						
	0	0	2	1	0	0	3
	0	0	2	1	0	1	4
	0	2	2	0	0	1	5

**6**	1	1	2	0	0	1	5
	1	1	2	0	0	1	5
	**LIFUP_1**						
	1	2	2	0	0	1	6
	4	5	0	0	0	1	10
	1	1	2	0	0	1	5
**7**							
	0	1	2	0	0	1	4
	0	1	2	1	0	1	5
	**LIFUP_1**						
	1	1	2	1	0	1	6
	0	1	2	1	0	1	5
	0	1	2	1	0	1	5
	0	1	2	1	0	1	5
	**LIFUP_2**						
	1	1	2	1	0	1	6
	1	1	2	1	0	1	6
	1	1	2	1	0	1	6
**8**	1	0	2	1	0	0	4
	1	0	2	1	0	0	4
	**LIFUP_1**						
	0	1	0	1	0	0	2
	0	1	2	1	0	0	4
**9**	1	0	1	1	0	2	5
	3	1	2	0	0	0	6
	**LIFUP_1**						
	1	2	1	1	0	1	6
	3	3	2	1	0	1	10
	3	3	2	1	0	1	10
**10**	1	1	2	0	0	1	5
	3	1	2	1	0	1	8
	**LIFUP_1**						
	0	1	2	1	0	0	4
	3	2	2	1	0	1	9
**11**	1	0	0	0	0	0	1
	0	0	1	0	0	0	1
	0	0	1	0	0	0	1
	**LIFUP_1**						
	0	0	1	1	0	0	2
	0	0	1	1	0	0	2

Supplementary Table S1. Here, all behavioral data is supplied for each subject. Though the number of assessments varies some between subjects, each subject received at least two CRS-R assessments prior to and following LIFU. These include, necessarily, 1 hour and 1 day prior to and following LIFU. Assessments are supplied chronologically. If additional assessments are present, they represent an assessment taken 1 week prior to and/or following LIFU.
